# Supplementary material for: Early formative objective structured clinical examinations for students in the pre-clinical years of medical education: A non-randomized controlled prospective pilot study
Source: PLoS One. 2023 Dec 7;18(12):e0294022. doi: 10.1371/journal.pone.0294022 (PMC10703252; doi:10.1371/journal.pone.0294022)
Supplement: S1 Appendix — (DOCX) [file pone.0294022.s001.docx]

**S1 Appendix. Details of the four formative OSCE stations**

**Session 1: Fever**

**INSTRUCTIONS TO STUDENTS**

You work in an emergency department and receive Jules, 16, who comes in with a fever. He comes to the emergency department unaccompanied. He is originally from the Ivory Coast and arrived in France three months ago. He has never been to your hospital and does not appear to have any medical follow-up.

You must carry out a complete interview of this patient and specify, in particular, the characteristics of the fever, as well as the associated signs.

You will be evaluated solely on your ability to conduct this interview.

You will not be asked to perform any clinical or complementary examinations.

**PATIENT INSTRUCTIONS**

**1/ initial information:**

You are Jules, 16 years old, and consulting alone for a fever. You are an unaccompanied minor who arrived in France three months ago and live in a hostel in precarious conditions.

Weight: 58 kg

Height: 178 cm.

You have no medical or surgical history. You have no notable family history and you have three sisters in good health in the Ivory Coast.

The candidate does not have to perform your physical examination during the interview. The consultation will be dedicated entirely to the interview. He/she should ask you about the characteristics of the fever and associated symptoms.

**2/ Data for the candidate (to be given to the candidate only if requested - Give if not asked after 5 minutes)**

Lifestyle, history, risk factors:

- Unaccompanied minor, arrived in France three months ago, lives in an unsanitary home (promiscuity with other occupants, poor ventilation, mold)

- You have no medical or surgical history

- You have no known allergies

- You are not taking any medication

- You do not smoke or drink

- No medical follow-up, unknown vaccination status, no health record, no growth chart available

Characteristics of the fever:

- Evolving over 4 weeks

- 38.5°C

- Daily, intermittent

- Mainly in the evening

- Measured in the axilla, corrected

- Gradual onset, no triggering factor found

Associated signs:

- Loss of 5 kg over the last 2 months*

- Significant asthenia for 2 months with anorexia

- Night sweats for 1 month

- Daily cough for 1 month,* with sputum, sometimes bloody

- Has felt a little breathless for 1 week

- Contagion: 1 occupant of the same dwelling has been coughing for 2 months, has not consulted a doctor.

***The fields marked with a star are to be specified to the candidate after 5 minutes if they are not asked spontaneously**

**GUIDELINES FOR THE EVALUATOR**

**1/ initial information**

You work in an emergency department and receive Jules, 16, who comes in with a fever. He comes to the emergency department unaccompanied. He is originally from the Ivory Coast and arrived in France three months ago. He has never been to your hospital and does not appear to have any medical follow-up.

The candidate does not have to perform a physical examination, nor propose any complementary examination during the interview. The consultation will be entirely dedicated to the interview. No diagnosis is expected at the end of this medical interview.

The simulated patient will be able to provide the data requested by the candidate.

**2/ objectives**

To assess the candidate's ability to conduct an interview.

To assess the candidate's ability to communicate with a patient in a respectful and comprehensible manner.

**NOTATION**

|  | Points |
| --- | --- |
| Knowing how to be/knowing how to do |  |
| Introduces self | 5 |
| Says Hello | 3 |
| Uses simple vocabulary | 3 |
| Allows the patient to express himself, listens to him, does not interrupt | 5 |
| Structured questioning | 3 |
|  |  |
| Interrogation |  |
| History and lifestyle: |  |
| Medical History | 5 |
| Surgical History | 1 |
| Familial History | 2 |
| Allergy | 1 |
| Treatments | 3 |
| Tobacco | 2 |
| Alcohol | 2 |
| Drug | 3 |
| Residence: apartment/house/household | 3 |
| Living conditions: crowded/number of occupants/unhealthy housing | 5 |
| Vaccination status | 4 |
| Health record | 2 |
|  |  |
| Characterization of fever |  |
| Since when | 10 |
| Temperature | 3 |
| Method for taking temperature | 2 |
| Gradual vs sudden onset | 2 |
| Daily or not | 4 |
| Plateau or intermittent | 2 |
| Timing of fever | 2 |
|  |  |
| Associated signs |  |
| Cough | 4 |
| Weight loss | 4 |
| Anorexia | 2 |
| Dyspnea | 2 |
| Night sweats | 1 |
| Counting | 10 |
|  | /100 |

**Session 2: Pelvic pain**

**INSTRUCTIONS TO STUDENTS**

You work in an emergency department and receive Miss C., 27, for pelvic pain. She thinks she is pregnant. She has not had any further tests. She works as a salesperson. She weighs 62 kg and measures 166 cm. Her only medical and surgical history is an appendectomy at the age of 13.

You must carry out a complete interview of this patient and, specify, in particular the characteristics of the pain, as well as the associated signs.

You will be evaluated solely on your ability to conduct this interview.

You will not be asked to perform any clinical or complementary examinations.

**PATIENT INSTRUCTIONS**

**1/ initial information:**

You are Miss C., 27 years old, and you are consulting for pelvic pain. You think you are pregnant. You have not had any additional tests. You work as a salesperson.

Weight: 62 kg

Height: 166 cm

Your only medical and surgical history is an appendectomy at the age of 13.

The candidate does not have to perform your physical examination during the interview. The consultation will be dedicated entirely to the interview. He/she should ask you about the characteristics of the pain and associated symptoms.

**2/ Data for the candidate (to be given to the candidate only if requested - Give if not asked after 5 minutes)**

- group A+

- you have no medical or surgical history other than appendicitis

- you have no known allergy

- you are not taking any medication

- you do not smoke

- You do not use any contraception

- You have a desire to become pregnant

- This is your first pregnancy

- Spontaneous pregnancy

- The date of your last menstrual period: // or 5SA

- You have not had an ultrasound or blood test

- You do not have any bleeding

- You have pain of low intensity (3/10) of progressive onset in the left iliac fossa, you have not taken painkillers, there has been no triggering factor in the last 48 hours, and there has been no calming of the pain.

- You have no fever or leucorrhea

- You have nausea

**GUIDELINES FOR THE EVALUATOR**

**1/ initial information:**

You work in an emergency department and receive Miss C., 27, for pelvic pain. She thinks she is pregnant. She has not had any further tests. She works as a salesperson. She weighs 62 kg and measures 166 cm. Her only medical and surgical history is an appendectomy at the age of 13.

The candidate does not have to perform a physical examination nor propose any complementary examination during the interview. The consultation will be entirely dedicated to the interview. No diagnosis is expected at the end of this medical interview.

The simulated patient will be able to provide the data requested by the candidate.

**2/ objectives**

To assess the candidate's ability to conduct an interview.

To assess the candidate's ability to communicate with a patient in a respectful and comprehensible manner.

**Notation**

|  | points |
| --- | --- |
| Knowing how to be/knowing how to do |  |
| Introduces self | 2 |
| Says Hello | 2 |
| Uses simple vocabulary | 1 |
| Allows the patient to express herself, listens to her, does not interrupt | 2 |
| Structured questioning | 4 |
| Interrogation |  |
| Usual treatment | 2 |
| Allergy | 2 |
| Blood type | 6 |
| Contraception | 6 |
| Desired pregnancy | 2 |
| Tobacco | 4 |
| History of ectopic pregnancy | 3 |
| Medically assisted procreation | 2 |
| Date of last period | 12 |
| Associated signs |  |
| Bleeding | 8 |
| Fever | 3 |
| Leukorrhea | 3 |
| Sympathetic signs of pregnancy | 4 |
| Pain | 8 |
| Characterization of pain |  |
| Triggering factor | 4 |
| Since when ? | 4 |
| Intensity | 4 |
| Aggravating/calming of pain | 4 |
| Location | 4 |
| Analgesics taken ? Effectiveness ? | 4 |
|  | /100 |

**Session 3: Anorexia Nervosa**

**INSTRUCTIONS TO STUDENTS**

You are a psychiatrist in private practice and receive Julie, 12 years old, with no previous history, accompanied by her parents for weight loss and eating difficulties. The parents tell you that they were referred by their general practitioner, who is concerned about Julie's weight loss. The blood test performed last week shows hypoalbuminemia. The rest of the workup is normal. After ruling out a digestive pathology, the general practitioner has referred Julie to you.

The clinical examination you performed today revealed amyotrophy, fine downy hair covering the entire body, and her hair is fragile and brittle. Her weight is 25 kg and her height 153 cm. Her heart rate is 62 bpm, blood pressure 110/80 mmHg, and body temperature 37.2°C. The rest of the clinical examination was unremarkable.

You must carry out a complete interrogation and formulate a diagnostic hypothesis to the child's parent.

You will be evaluated solely on your ability to conduct this interview.

You will not be asked to perform any clinical or complementary examinations.

**PATIENT INSTRUCTIONS**

**1/ initial information:**

You are the parent of Julie, who is 12 years old. You are accompanying her for a psychiatric evaluation on referral from your general practitioner. You tell the psychiatrist that Julie's weight loss has been progressing for several months and that she has cut many foods from her diet. You are concerned because the weight loss has accelerated since your last GP appointment last week.

You have forgotten the health booklet but you have height-weight curves made by your general practitioner (**Wait for the student to ask for the health record to give the curves, if the student does not ask for the health record or the curves by 4 minutes, offer them spontaneously)**

**2/ Data for the candidate (to be given to the candidate only if requested - Give if not asked after 5 minutes)**

| Health booklet | You forgot it, give the height-weight curves |
| --- | --- |
| Family History | One of Julie's aunts has always been very thin and is very careful with her diet |
| Consanguinity | No |
| Siblings | First child, had a 9-year-old brother who is doing well |
| Antenatal | Normal pregnancy, antenatal ultrasound: normal |
| Delivery | Full-term vaginal delivery, no complications |
| Early dietary history | Breastfeeding the first 2 months, no sucking difficulties, regular weight gain, no difficulties with diversification, no GERD. Eating at the canteen since preschool, no difficulties reported. |
| Early development | First steps at 13 months, first words at 12 months, no developmental delay |
| Personal History | None, no digestive pathology |
| Vaccination | Vaccinations are up to date |
| Schooling | Excellent student, finds it insufficient to have a 17 average, works later and later at night. No decrease in grades, no concentration difficulties reported. |
| Leisure | Intensive gymnastics practice 3 hours during the week and competition on weekends |
| Triggering factor | A friend from the gym told her she wasn't going to fit into her leotard anymore |
| Feeding | For the last 6 months, Julie has become highly vigilant about her diet. She checks food packaging and has progressively eliminated sweet foods, whereas she was greedy before. She refuses to use butter or oil.  The situation has worsened over the last few weeks. She refuses to eat breakfast and the school has alerted her parents because Julie only eats her bread in the canteen. For the afternoon snack, she is satisfied with an apple and a glass of water at 4pm. Her parents have to argue with her to get her to eat a little rice and ham in the evening. They have to watch her so that she does not throw her meal in the garbage. |
| Weight loss | Maximum weight reached 6 months ago: 34 kg. Note that she was 27.5 kg last week at her GP's (-2.5 kg in 1 week). |
| Physical activity | Julie has started exercising on the nights she does not have gym class. She prefers to stand in front of the television.  On weekends, she walks for several hours under the pretext of walking the dog, even though she had not taken much care of it before. |
| Associated symptoms | No vomiting.  Julie complains of having a stomachache after meals and feeling bloated.  Julie spends a lot of time looking at herself in the mirror or in the reflection of windows and only wears large sweaters that hide her shape.  Not yet regular (mother's period: 10 years) |
| Thymic assessment | Julie does not have any particular thymic disorder |
| **After 4 minutes** | **If the candidate seems disoriented in his diagnostic approach (does not ask for the health record or the height-weight curves), spontaneously propose the curves.** |
| If candidate does not spontaneously explain the curves to you | Ask him to explain the BMI and height-weight curves |
| **After 6 minutes** | **You should ask for his or her diagnostic hypothesis** |

|  | |  |
| --- | --- | --- |
| Presentation | (3) |  |
| Let the parent express the reason for coming without interrupting | 3 |  |
| Interview to help with the diagnostic approach | (50) |  |
| History of pregnancy and early development | 3 |  |
| Seeks family history of feeding disorders and personal medical history | 2 | 4 |
| Ask for the last weight | 10 |  |
| Ask about purging behaviors | 5 |  |
| Ask about a trigger/beginning factor of the disorders | 5 |  |
| Ask about knowledge of anorexea | 10 |  |
| Ask about any increase in physical activity | 10 |  |
| Evaluate the impact on schooling and functioning | 3 |  |
| Diagnostic approach | (30) |  |
| Spontaneously asks for the health record/growth curves + Calculates the current BMI | 10 | 15 |
| Spontaneously explains the curves to the parent + talks about a break in the height growth curve | 5 | 15 |
| Diagnosis evoked | (10) |  |
| Diagnosis of anorexia nervosa | 10 |  |
| Communication skills | (7) |  |
| Ability to conduct the interview and organize the questioning | 7 |  |
| Total/100 |  |  |

**Height-weight curves**


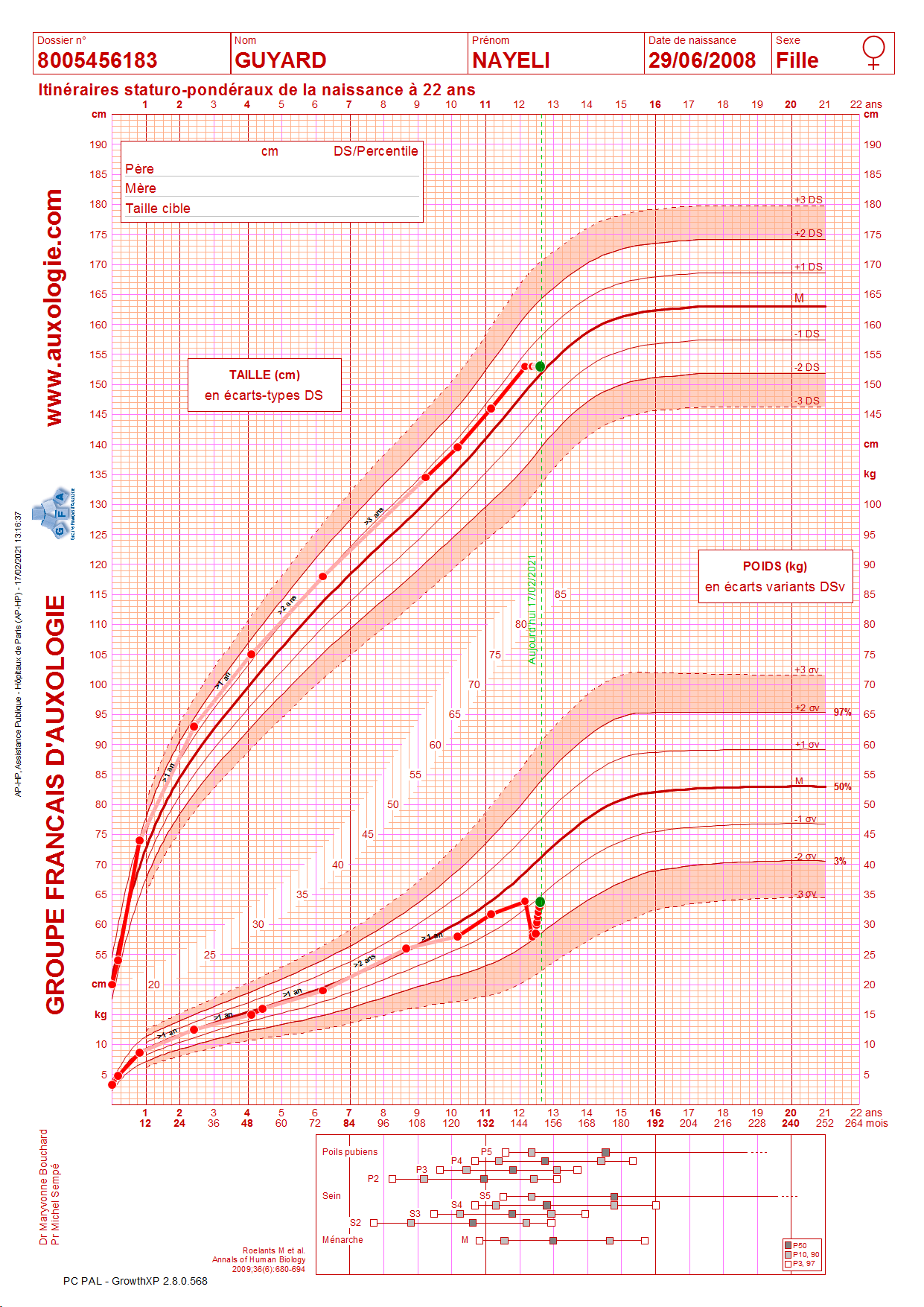

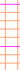

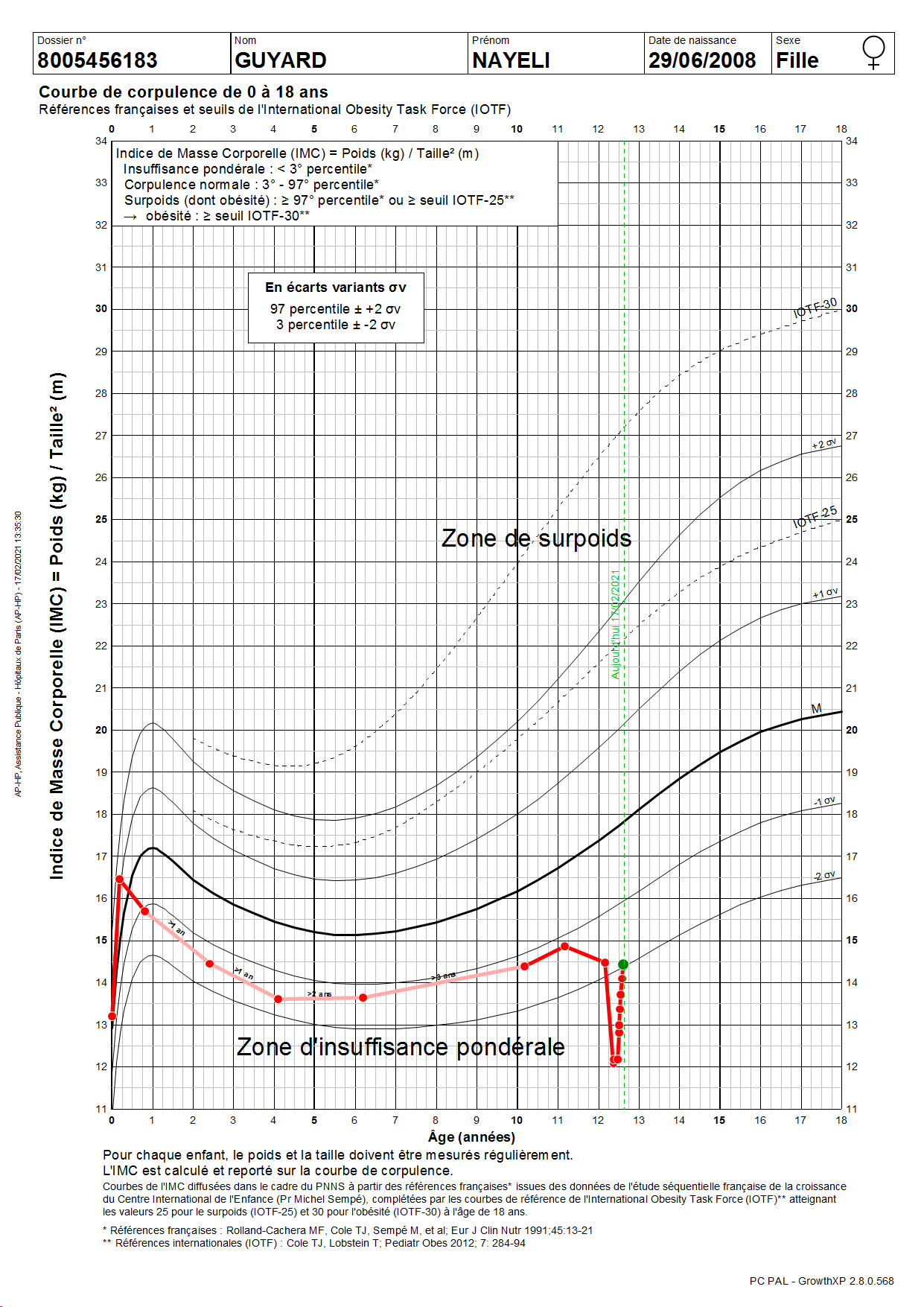

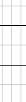


**Session 4: Headaches**

**INSTRUCTIONS TO STUDENTS**

You work in an emergency department and receive Thea, 10 years old, who comes in for a headache. She comes to the emergency room accompanied by her parents. She has never been hospitalized or operated on and has no allergies. She has never been to the hospital before.

You must carry out a complete interrogation of this patient.

You will be evaluated solely on your ability to conduct this interview. You will not be asked to perform any clinical or complementary examinations.

**PATIENT INSTRUCTIONS**

**1/ initial information**

You are Thea, 10 years old, and you are consulting for a headache. You have no medical or surgical history. You are accompanied by your parents.

The candidate does not have to perform your physical examination during the interview. The consultation will be dedicated entirely to the interview. He/she should ask you about the characteristics of the fever and associated symptoms.

**2/ Data for the candidate (to be given to the candidate only if requested - Give if not asked after 5 minutes)**

Tell the candidate that the treating physician had mentioned migraines and ask if this diagnosis is plausible.**GUIDELINES FOR THE EVALUATOR**

**1/ initial information**

You work in an emergency department and receive Thea, 10 years old, who comes in for a headache. She comes to the emergency room accompanied by her parents. She has never been hospitalized or operated on and has no allergies. She has never been to the hospital before.

The candidate must carry out a complete interrogation of this patient

The candidate will be evaluated solely on his/her ability to conduct this interview. The candidate will not be asked to perform any clinical or complementary examinations.

**2/ objectives**

To assess the candidate's ability to conduct an interview

To assess the candidate's ability to communicate with a patient in a respectful and comprehensible manner.

**NOTATION**

|  | Answer | Points |
| --- | --- | --- |
| **Knowing how to be/knowing how to do** |  |  |
| Introduces self |  | 3 |
| Says Hello |  | 3 |
| Uses simple vocabulary |  | 3 |
| Allows the patient to express herself, listens to her, does not interrupt |  | 3 |
| Structured questioning |  | 5 |
| **Interrogation** |  |  |
| Previous episode/first episode | 6th episode | 3 |
| Date of the first episode | 2 months ago | 3 |
| Current episode identical to the other episodes | Yes | 4 |
| Trigger factor | None | 3 |
| Installation time | A few minutes | 4 |
| Localization of pain | Temporal | 4 |
| Unilateral/bilateral character | Unilateral | 4 |
| Always on the same side | No | 3 |
| Continuous/pulsating character | Pulsating | 4 |
| Intensity | High | 5 |
| Positionality | No | 3 |
| Phonophobia | Yes | 3 |
| Photophobia | Yes | 3 |
| Increase in effort (climbing stairs...) | Yes | 5 |
| Nausea and/or vomiting | No | 5 |
| Aura | No | 3 |
| Fever | No | 5 |
| Duration of headaches | A few hours | 5 |
| Treatments used | Paracetamol | 5 |
| Effectiveness | Not very effective | 3 |
| Impact on the activities of daily life (school absenteeism...) | None | 3 |
| Brain imagery performed | No | 3 |
|  |  | /100 |
