# Supplementary material for: Early formative objective structured clinical examinations for students in the pre-clinical years of medical education: A non-randomized controlled prospective pilot study
Source: PLoS One. 2023 Dec 7;18(12):e0294022. doi: 10.1371/journal.pone.0294022 (PMC10703252; doi:10.1371/journal.pone.0294022)
Supplement: S2 Appendix — (DOCX) [file pone.0294022.s002.docx]

**S2 Appendix. Questionnaire administered to students from the intervention group.**

1. **Regarding your opinion on the formative OSCE sessions, do you agree with the following:**

|  | Fully agree | Agree | No opinion | Disagree | Fully disagree |
| --- | --- | --- | --- | --- | --- |
| 1) The OSCE sessions were useful |  |  |  |  |  |
| 2) The individual debriefing was useful |  |  |  |  |  |
| 3) The collective debriefing was useful |  |  |  |  |  |
| 4) The formative OSCEs were stressful |  |  |  |  |  |
| 5) The formative OSCEs were adapted to the student’s knowledge and skills |  |  |  |  |  |

1. **Regarding your opinion on the summative OSCE sessions, do you agree with the following:**

|  | Fully agree | Agree | No opinion | Disagree | Fully disagree |
| --- | --- | --- | --- | --- | --- |
| 6) The formative OSCEs reduced the level of stress in subsequent summative OSCEs |  |  |  |  |  |
| 7) The formative OSCEs improved skills in subsequent summative OSCEs |  |  |  |  |  |

1. **Regarding the organization of the formative OSCE sessions**

8) The OSCE scenario was immersive: □ Yes □ No

9) The scenario was immersive with medical teachers simulating the patient/parent: □ Yes

□ No

10) The scenario was immersive with medical teachers being the evaluators: □ Yes □ No

11) The number of four formative OSCE sessions was: □ Too few □ Suitable □ Too many.

You can add free comments on the formative OSCEs:
