# Supplementary material for: Early formative objective structured clinical examinations for students in the pre-clinical years of medical education: A non-randomized controlled prospective pilot study
Source: PLoS One. 2023 Dec 7;18(12):e0294022. doi: 10.1371/journal.pone.0294022 (PMC10703252; doi:10.1371/journal.pone.0294022)
Supplement: S1 Fig — (DOCX) [file pone.0294022.s003.docx]

**S3 Figure. Results of the questionnaire administered to intervention group students, N = 17.**

1. **The OSCE sessions were useful**


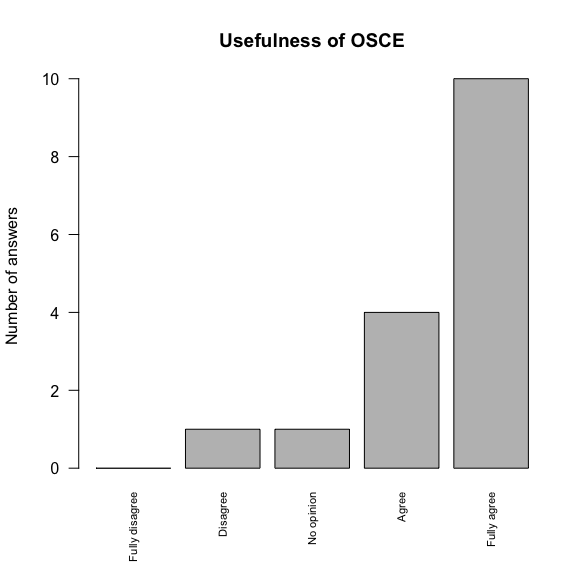


OSCE: objective structured clinical examination

1. **The individual debriefing was useful**

**
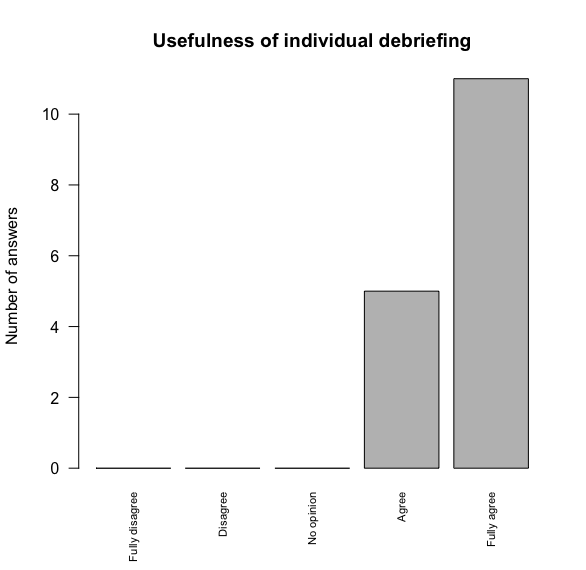
**

1. **The collective debriefing was useful**


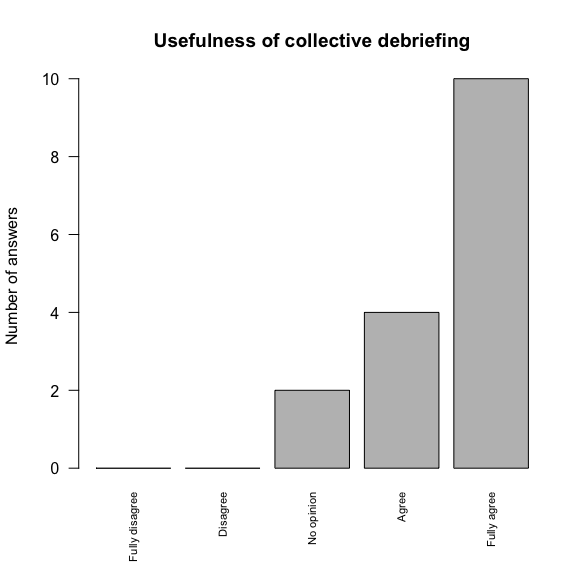


1. **The formative OSCEs were stressful**


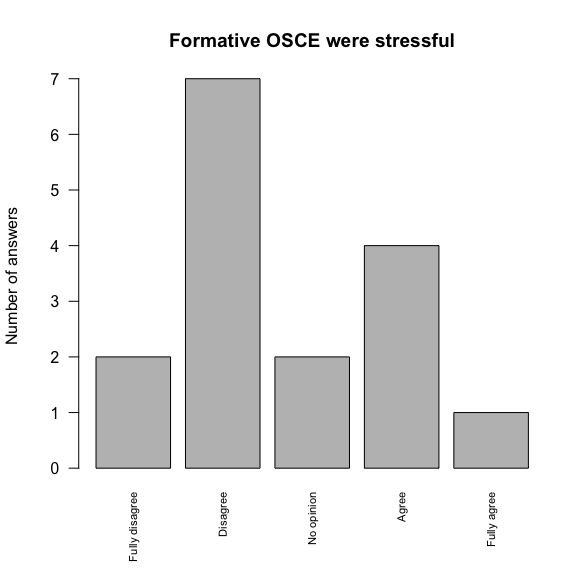


1. **The formative OSCEs were adapted to student’s knowledge and skills**

**
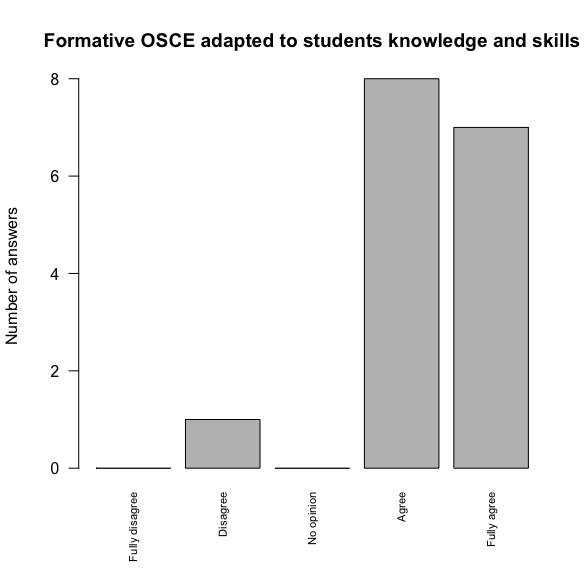
**

1. **The formative OSCEs reduced the level of stress in subsequent summative OSCEs**

**
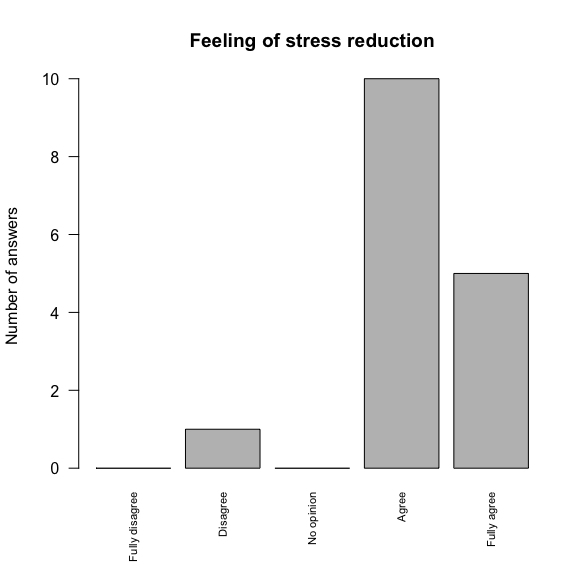
**

1. **The formative OSCEs improved skills for subsequent summative OSCEs**

**
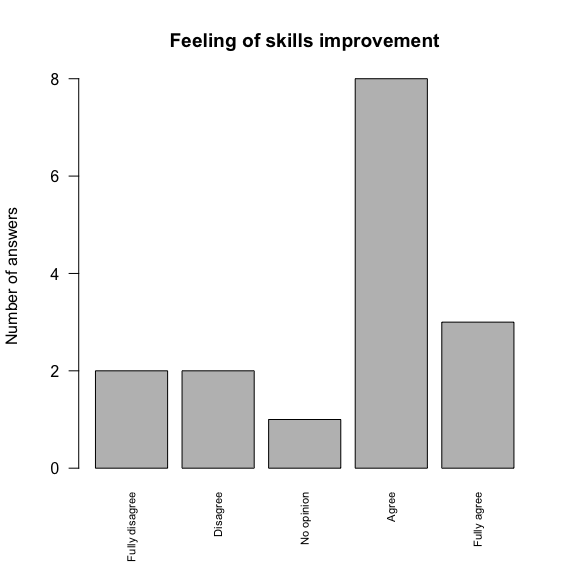
**

1. **The OSCE scenario was immersive**

**
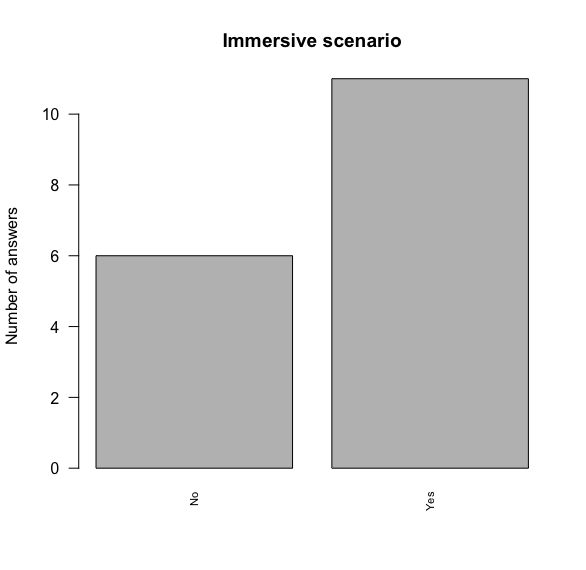
**

1. **The scenario was immersive with medical teachers simulating the patient/parent**

**
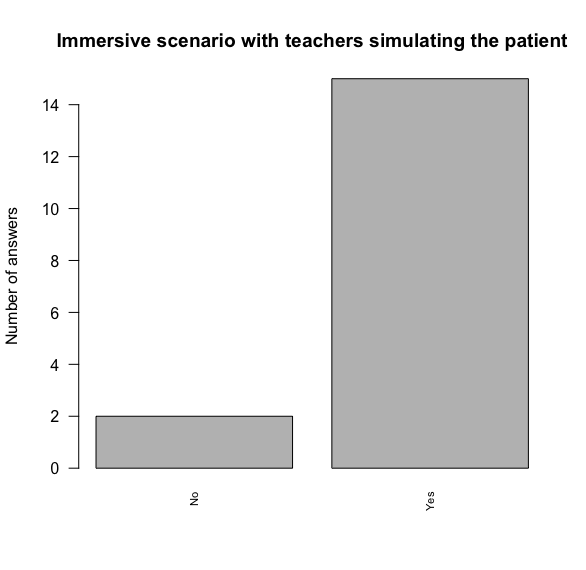
**

1. **The scenario was immersive with medical teachers being the evaluators**

**
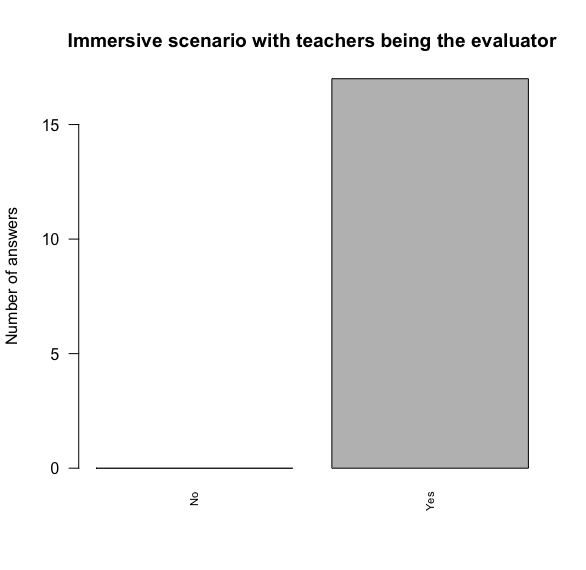
**

1. **The number of four formative OSCE sessions was:**

**
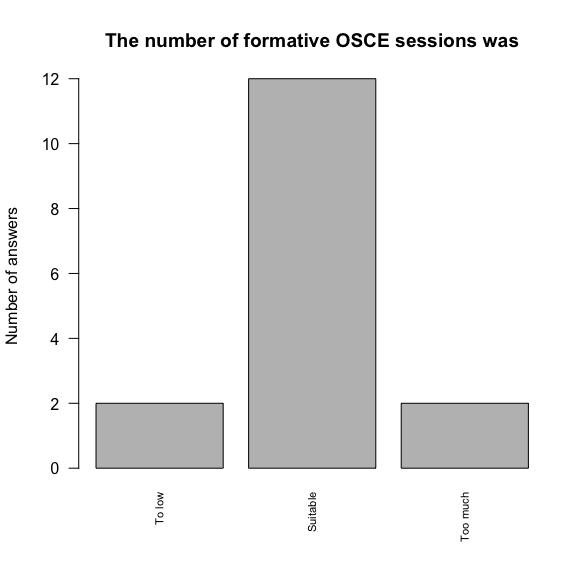
**
