# Supplementary material for: Early formative objective structured clinical examinations for students in the pre-clinical years of medical education: A non-randomized controlled prospective pilot study
Source: PLoS One. 2023 Dec 7;18(12):e0294022. doi: 10.1371/journal.pone.0294022 (PMC10703252; doi:10.1371/journal.pone.0294022)
Supplement: Attachment — Submitted filename: Response to Reviewers.docx [file pone.0294022.s004.docx]

Naïm Ouldali, MD, PhD

General Pediatrics, infectious diseases and internal medicine department

Robert Debré University Hospital

48 Bd Sérurier, 75019 Paris, France.

Email: [naim.ouldali@aphp.fr](mailto:naim.ouldali@aphp.fr).

Phone: 0033140032048

Ayse Hilal Bati

Academic Editor

PLOS ONE

Paris, October 11^th^, 2022

Re-submission of manuscript PONE-D-22-23055 entitled: “***Early formative objective structured clinical examinations for students in the pre-clinical years of medical education: a non-randomized controlled prospective pilot study”***

Dear Editor,

We would like to thank you for giving us the opportunity to submit the revised manuscript entitled *“****Early formative objective structured clinical examinations for students in the pre-clinical years of medical education: a non-randomized controlled prospective pilot study****”*. We took into account the reviewers’ comments and suggestions and modified the manuscript accordingly.

You will find below the point-by-point responses outlining the modifications made to the manuscript and the additional analyses performed. As requested, we have submitted the revised manuscript incorporating the changes below, together with a marked-up copy of the changes made from the previous article as tracked changes.

We hope that this revised and improved version of our manuscript will be suitable for publication in *PLOS ONE.*

Sincerely,

Naïm Ouldali, MD, PhD.

PONE-D-22-23055

Early formative objective structured clinical examinations for students in the pre-clinical years of medical education: a non-randomized controlled prospective pilot study.

PLOS ONE

Dear Dr. Ouldali,

Thank you for submitting your manuscript to PLOS ONE. After careful consideration, we feel that it has merit but does not fully meet PLOS ONE’s publication criteria as it currently stands. Therefore, we invite you to submit a revised version of the manuscript that addresses the points raised during the review process.

==============================

I am happy to have been involved in the evaluation process of this article. It is important that article authors are educators working in clinical sciences. Because this type of research is generally in the field of interest of educators working in Medical Education departments.

I think that there are aspects that are not clear enough in the article in terms of the fact that the research topic is remarkable and reader expectations about the subject. Some of these are the extreme difference between the number of students in the case and control groups, and the inadequacy of explanation for qualitative research, which is especially mentioned in the reviewer suggestions.

- First, we would like to thank the editor and the reviewers for their careful review and their important suggestions provided to improve the manuscript.
- We fully agree that the difference between the number of students in the two groups is an issue. As suggested and to address this, we performed an additional analysis by randomly selecting 80 students among the control group, thus providing a 1:4 ratio between the intervention and control groups, and reanalyzed the main outcome among this sub-group.

We performed this process 5 times, and in all cases, the results remained very similar.

- This additional analysis is now included in the revised manuscript (appendix 3). The following sentences have been added:
  - Method section line 182: “To deal with the difference in sample size between the intervention and the control group, we also conducted sub-group analyses by randomly selecting 80 students among the control group, thus providing a 1:4 ratio between the intervention and control groups to reanalyze the main outcome. This process was performed 5 times, to explore whether the difference in sample size between the two groups could have influenced our findings.”
  - Results section, line 234: “Similar results were obtained when conducting multivariate linear regression adjusted for age, repetition of the past year, and the number of students retaking exams (Table 1), or when conducting subgroup analyses with a 1:4 ratio between the intervention and the control group (appendix 3).”
  - Discussion section, line 335: “This study had several limitations. First, our intervention group could be considered as being small (20 students). However, it was calculated a priori, to have sufficient power to detect a potential difference between groups, and subgroup analyses with a 1:4 ratio between the intervention and control groups found unchanged results.”

The fact that the research was conducted with a mixed methodology necessitates detailed explanations about the methodology. Particular attention should be paid to clarity and detailed expression as a criterion of validity and reliability in the qualitative dimension.

- We thank the editor and the reviewer #2 for this important comment and suggestion. As requested, we clarified the method section reporting the qualitative study, to avoid misunderstanding regarding the qualitative method used (participative observation of the formative OSCE program and not formal interviews). As suggested by the reviewer #2 and the editor, the following changes were made:
  - Method section, line 160: “In addition, to complete the evaluation of this early OSCE training program, a qualitative study was conducted via the observation of five of the formative OSCE sessions (the four sessions of one subgroup and one session of another, convenience sampling). Marshall and Rossman defined observation as "the systematic description of events, behaviors, and artifacts in the social setting chosen for study" [13]. This involves active looking, informal interviewing, and writing detailed field notes [14]. Two independent public health researchers trained in qualitative analysis (EL and CE) took extensive notes on students’ behavior and attitudes during observation and did informal interviews with students before and after the sessions. Mixed methods were used in this study for triangulation purposes, in order to converge results from different methods with the goal of validating interpretations.”
  - Method section, line 191: “Inductive thematic analysis was used for qualitative analysis. Thus, we coded the data without trying to fit it into a preexisting coding frame. The analysis was data-driven [15].”
  - Results section, line 253: “The main themes that emerged from the observation analysis were related to stress, the ease and difficulties of the students to act as expected during the exercise, and the reported and observed benefits.”

For the topic covered in the article, I would expect different training methods, such as training with simulated patients, to at least be included in the discussion.

- We fully agree with the editor. As suggested, to better highlight this point, the following changes were made:
  - Discussion section line 342: “Third, formative OSCE sessions were performed by medical personal known by students rather than standardized simulated patients. This could lead to a loss of credibility during the OSCE. However, a recent study showed that known medical examiners were perceived to be more credible than standardized patients [17]. One explanation relies on social identity theory, which suggests that students identified known medical personal as their ‘in-group’ versus the ‘‘out-group’’ for standardized patients, influencing the perception of each group [18]. Further studies are required to compare the impact of different formative programs using standardized simulated patients.”

Therefore, I think it would be appropriate to correct the article.

- We thank the editor for giving us the opportunity to correct the article, and hope that the changes provided are suitable. We are looking forward to a continued discussion for any remaining issues or additional questions.

==============================

Please submit your revised manuscript by Nov 03 2022 11:59PM. If you will need more time than this to complete your revisions, please reply to this message or contact the journal office at [plosone@plos.org](mailto:plosone@plos.org). When you're ready to submit your revision, log on to <https://www.editorialmanager.com/pone/> and select the 'Submissions Needing Revision' folder to locate your manuscript file.

Please include the following items when submitting your revised manuscript:

- A rebuttal letter that responds to each point raised by the academic editor and reviewer(s). You should upload this letter as a separate file labeled 'Response to Reviewers'.
- A marked-up copy of your manuscript that highlights changes made to the original version. You should upload this as a separate file labeled 'Revised Manuscript with Track Changes'.
- An unmarked version of your revised paper without tracked changes. You should upload this as a separate file labeled 'Manuscript'.

If you would like to make changes to your financial disclosure, please include your updated statement in your cover letter. Guidelines for resubmitting your figure files are available below the reviewer comments at the end of this letter.

If applicable, we recommend that you deposit your laboratory protocols in [protocols.io](http://protocols.io) to enhance the reproducibility of your results. [Protocols.io](http://Protocols.io) assigns your protocol its own identifier (DOI) so that it can be cited independently in the future. For instructions see: [https://journals.plos.org/plosone/s/submission-guidelines#loc-laboratory-protocols](http://antiphishing.aphp.fr/v4?f=QzZIM281ODAzYTI3VWdDZ2CoG3xmuz9D01DRNMertR0LmeIuh029luQe0XiIRvFotTv_x2pcDS4W6TP-83OaDA&i=ZWZlVDl1bGVTN25wejkxNJlt_xIxAU6PfyIczIyxTmY&k=lrFr&r=dHhXakJUYm9oMzROM253NkbMcU6yq7tK3Maq3fgh6fYUncbCsLbX6vKrChgC-jyJ&s=02cf96e295c8e24f992bcbbd229ab787a7e89907a132e9014a4196893d6f0984&u=https%3A%2F%2Fjournals.plos.org%2Fplosone%2Fs%2Fsubmission-guidelines%23loc-laboratory-protocols). Additionally, PLOS ONE offers an option for publishing peer-reviewed Lab Protocol articles, which describe protocols hosted on [protocols.io](http://protocols.io). Read more information on sharing protocols at [https://plos.org/protocols?utm_medium=editorial-email&utm_source=authorletters&utm_campaign=protocols](http://antiphishing.aphp.fr/v4?f=QzZIM281ODAzYTI3VWdDZ2CoG3xmuz9D01DRNMertR0LmeIuh029luQe0XiIRvFotTv_x2pcDS4W6TP-83OaDA&i=ZWZlVDl1bGVTN25wejkxNJlt_xIxAU6PfyIczIyxTmY&k=lrFr&r=dHhXakJUYm9oMzROM253NkbMcU6yq7tK3Maq3fgh6fYUncbCsLbX6vKrChgC-jyJ&s=8a7ed11d7e7daf01ced53bc7b362479216f1500dd31b0f3840d0fd95432d08b7&u=https%3A%2F%2Fplos.org%2Fprotocols%3Futm_medium%3Deditorial-email%26utm_source%3Dauthorletters%26utm_campaign%3Dprotocols).

We look forward to receiving your revised manuscript.

Kind regards,

Ayse Hilal Bati, Associate Professor
Academic Editor
PLOS ONE

Journal requirements:

When submitting your revision, we need you to address these additional requirements.

1.  Please ensure that your manuscript meets PLOS ONE's style requirements, including those for file naming. The PLOS ONE style templates can be found at

[https://journals.plos.org/plosone/s/file?id=wjVg/PLOSOne_formatting_sample_main_body.pdf](http://antiphishing.aphp.fr/v4?f=QzZIM281ODAzYTI3VWdDZ2CoG3xmuz9D01DRNMertR0LmeIuh029luQe0XiIRvFotTv_x2pcDS4W6TP-83OaDA&i=ZWZlVDl1bGVTN25wejkxNJlt_xIxAU6PfyIczIyxTmY&k=lrFr&r=dHhXakJUYm9oMzROM253NkbMcU6yq7tK3Maq3fgh6fYUncbCsLbX6vKrChgC-jyJ&s=9e254c4b89b55fba760ba0cbcdee73c6731e9720372fb98b3208eae16f88e149&u=https%3A%2F%2Fjournals.plos.org%2Fplosone%2Fs%2Ffile%3Fid%3DwjVg%2FPLOSOne_formatting_sample_main_body.pdf)  and

[https://journals.plos.org/plosone/s/file?id=ba62/PLOSOne_formatting_sample_title_authors_affiliations.pdf](http://antiphishing.aphp.fr/v4?f=QzZIM281ODAzYTI3VWdDZ2CoG3xmuz9D01DRNMertR0LmeIuh029luQe0XiIRvFotTv_x2pcDS4W6TP-83OaDA&i=ZWZlVDl1bGVTN25wejkxNJlt_xIxAU6PfyIczIyxTmY&k=lrFr&r=dHhXakJUYm9oMzROM253NkbMcU6yq7tK3Maq3fgh6fYUncbCsLbX6vKrChgC-jyJ&s=b1c412ea2411872a8ad7024e04e0b61d113fcab209dd693eb00da8ce97fa64f3&u=https%3A%2F%2Fjournals.plos.org%2Fplosone%2Fs%2Ffile%3Fid%3Dba62%2FPLOSOne_formatting_sample_title_authors_affiliations.pdf)

- Done

2. We note that you have indicated that data from this study are available upon request. PLOS only allows data to be available upon request if there are legal or ethical restrictions on sharing data publicly. For more information on unacceptable data access restrictions, please see <http://journals.plos.org/plosone/s/data-availability#loc-unacceptable-data-access-restrictions>.

In your revised cover letter, please address the following prompts:

a) If there are ethical or legal restrictions on sharing a de-identified data set, please explain them in detail (e.g., data contain potentially sensitive information, data are owned by a third-party organization, etc.) and who has imposed them (e.g., an ethics committee). Please also provide contact information for a data access committee, ethics committee, or other institutional body to which data requests may be sent.

b) If there are no restrictions, please upload the minimal anonymized data set necessary to replicate your study findings as either Supporting Information files or to a stable, public repository and provide us with the relevant URLs, DOIs, or accession numbers. For a list of acceptable repositories, please see <http://journals.plos.org/plosone/s/data-availability#loc-recommended-repositories>.

We will update your Data Availability statement on your behalf to reflect the information you provide.

- We thank the editor for this remark. Indeed, the data that support the findings are owned by a third-party organization (the Paris University OSCE group), but can be available upon request. As suggested, we have updated this paragraph in the manuscript accordingly.

3. Please include captions for your Supporting Information files at the end of your manuscript, and update any in-text citations to match accordingly. Please see our Supporting Information guidelines for more information: <http://journals.plos.org/plosone/s/supporting-information>.

- Done

Additional Editor Comments :

Dear Author/s,
I am happy to have been involved in the evaluation process of this article. It is important that article authors are educators working in clinical sciences. Because this type of research is generally in the field of interest of educators working in Medical Education departments. I think that there are aspects that are not clear enough in the article in terms of the fact that the research topic is remarkable and reader expectations about the subject. Some of these are the extreme difference between the number of students in the case and control groups, and the inadequacy of explanation for qualitative research, which is especially mentioned in the reviewer suggestions. The fact that the research was conducted with a mixed methodology necessitates detailed explanations about the methodology. Particular attention should be paid to clarity and detailed expression as a criterion of validity and reliability in the qualitative dimension.
For the topic covered in the article, I would expect different training methods, such as training with simulated patients, to at least be included in the discussion.
Therefore, I think it would be appropriate to correct the article.


[Note: HTML markup is below. Please do not edit.]

Reviewers' comments:

Reviewer's Responses to Questions

**Comments to the Author**

1. Is the manuscript technically sound, and do the data support the conclusions?

The manuscript must describe a technically sound piece of scientific research with data that supports the conclusions. Experiments must have been conducted rigorously, with appropriate controls, replication, and sample sizes. The conclusions must be drawn appropriately based on the data presented.

Reviewer #1: Yes

Reviewer #2: Yes

2. Has the statistical analysis been performed appropriately and rigorously?

Reviewer #1: I Don't Know

Reviewer #2: Yes

3. Have the authors made all data underlying the findings in their manuscript fully available?

The [PLOS Data policy](http://antiphishing.aphp.fr/v4?f=QzZIM281ODAzYTI3VWdDZ2CoG3xmuz9D01DRNMertR0LmeIuh029luQe0XiIRvFotTv_x2pcDS4W6TP-83OaDA&i=ZWZlVDl1bGVTN25wejkxNJlt_xIxAU6PfyIczIyxTmY&k=lrFr&r=dHhXakJUYm9oMzROM253NkbMcU6yq7tK3Maq3fgh6fYUncbCsLbX6vKrChgC-jyJ&s=8abf6d97b276600ea02c4b10b041732e5fc2af10ada5807fa1d00bab718729c0&u=http%3A%2F%2Fwww.plosone.org%2Fstatic%2Fpolicies.action%23sharing) requires authors to make all data underlying the findings described in their manuscript fully available without restriction, with rare exception (please refer to the Data Availability Statement in the manuscript PDF file). The data should be provided as part of the manuscript or its supporting information, or deposited to a public repository. For example, in addition to summary statistics, the data points behind means, medians and variance measures should be available. If there are restrictions on publicly sharing data—e.g. participant privacy or use of data from a third party—those must be specified.

Reviewer #1: No

Reviewer #2: Yes

4. Is the manuscript presented in an intelligible fashion and written in standard English?

PLOS ONE does not copyedit accepted manuscripts, so the language in submitted articles must be clear, correct, and unambiguous. Any typographical or grammatical errors should be corrected at revision, so please note any specific errors here.

Reviewer #1: Yes

Reviewer #2: Yes

5. Review Comments to the Author

Please use the space provided to explain your answers to the questions above. You may also include additional comments for the author, including concerns about dual publication, research ethics, or publication ethics. (Please upload your review as an attachment if it exceeds 20,000 characters)

Reviewer #1: This simple study takes the hypothesis that a formative assessment task prior to a summative assessment task is likely to result in increased performance in that summative task. Authors use some nice techniques to show that the effect is limited to tasks specifically included in the formative task and for the intervention group. They claim the study is sufficiently powered despite the large discrepancy in case vs control group sizes. Whilst there have been similar studies in the literature over the years, the authors are keen to make a distinction between this study in the pre-clinical years, albeit focusing on a clinical skill (history-taking), and other studies based in clinical years. I feel this is stretched in this day, since many medical programs have long since mixed clinical work with clinical science learning. Nonetheless there is a contribution to the literature here.

- We thank the reviewer for this comment and these suggestions.
- We fully agree that the difference between the number of students in the two groups is an issue. As suggested, to address this, we performed an additional analysis by randomly selecting 80 students among the control group, thus providing a 1:4 ratio between the intervention and control groups, and reanalyzed the main outcome among this sub-group.

We performed this process 5 times, and in all cases, the results remained very similar.

- This additional analysis is now included in the revised manuscript (appendix 3). The following sentences have been added:
  - Method section line 182: “To deal with the difference in sample size between the intervention and the control group, we also conducted sub-group analyses by randomly selecting 80 students among the control group, thus providing a 1:4 ratio between the intervention and control groups to reanalyze the main outcome. This process was performed 5 times, to explore whether the difference in sample size between the two groups could have influenced our findings.”
  - Results section, line 234: “Similar results were obtained when conducting multivariate linear regression adjusted for age, repetition of the past year, and the number of students retaking exams (Table 1), or when conducting subgroup analyses with a 1:4 ratio between the intervention and the control group (appendix 3).”
  - Discussion section, line 335: “This study had several limitations. First, our intervention group could be considered as being small (20 students). However, it was calculated a priori, to have sufficient power to detect a potential difference between groups. Subgroup analyses with a 1:4 ratio between the intervention and control groups found unchanged results.”
- Regarding the distinction between preclinical and clinical years, we fully agree with the reviewer that some medical teaching programs now include clinical work early in the medical program. To better discuss this point, the following changes were made:
  - Discussion section line 297: “As several universities started to introduce clinical work earlier in the medical curriculum [8–10], this may also ease OSCE implementation at the early stage of the medical teaching program."

1. The study presents the results of original research.
Yes
2. Results reported have not been published elsewhere, to the best of my knowledge.
Yes
3. Experiments, statistics, and other analyses are performed to a high technical standard and are described in sufficient detail.
I am not an expert in statistical analysis but the analysis appears cogent from my perspective.
4. Conclusions are presented in an appropriate fashion and are supported by the data.
Yes
5. The article is presented in an intelligible fashion and is written in standard English.
Yes - there are some minor typos but nothing more e.g. personal used instead of personnel.
6. The research meets all applicable standards for the ethics of experimentation and research integrity.
Yes
7. The article adheres to appropriate reporting guidelines and community standards for data availability.
The authors state they will provide data but seeing that I am not a statistical expert that would not help in interpretation.

- We thank the reviewer for these positive comments.

Reviewer #2: Thank-you for submitting this manuscript which is very well written and reports an interesting study.
My only concern regards the qualitative component of the work, which I do not think is presented in adequate detail.
This needs to be addressed with regard to the following issues:
- 1. It is stated on page 12, lines 238-240 that the main themes that emerged from the qualitative analysis were related to....."
Were these the actual main themes? If so this should be made clear. Were there any sub-themes?
- 2. More detail is needed regarding data collection. How many interviews were conducted with how many students? What questions were asked? In what format were the interview? Where were they conducted? Were they recorded?
-3. Likewise how the data analyses was conducted need further explanation. Were the interviews audio recorded and transcribed, for example? If so, by whom? How were the themes decided on?
-4. It would also be useful for some quotes from the students to be included to illustrate/ support the main themes.

If these issues cannot be addressed, I recommend removing this entire section and reference to it from the paper.

- We thank the reviewer for these comments and questions which prompted us to clarify the use of qualitative methods in our article. We did not carry out formal interviews, the qualitative data were collected from observations and informal exchanges that took place before and after the formative OSCE sessions.
- Participant observation has been a hallmark of both anthropological and sociological studies for many years. In recent years, the field of education has seen an increase in the number of qualitative studies that include participant observation as a way to collect information (*Kawulich, 2005*). Marshall and Rossman defined observation as "the systematic description of events, behaviors, and artifacts in the social setting chosen for study" (*Marshall, 1989)*. Observations enable the researcher to describe existing situations using the five senses, providing a "written photograph" of the situation under study (*Erlandson, 1993).* It involves active looking, informal interviewing, and detailed field notes writing *(DeWalt, 2002).* DeWalt and DeWalt (2002) suggested that participant observation should be used as a way to increase the validity of the study, as observations may help the researcher have a better understanding of the context and phenomenon under study. Validity is stronger with the use of additional strategies used with observation, such as quantitative methods – in our study, questionnaires *(DeWalt, 2002).* All these reasons led us to choose this qualitative research method to ensure the description of our educational innovation and better interpret the results of the quantitative analysis.
- When writing up one's ethnographic observations, it is advised to follow the lead of Spradley & McCurdy (1972) and find a cultural scene, spend time with the informants, asking questions and clarifying answers, analyze the material, pulling together the themes into a well-organized story. We therefore carried out an inductive thematic analysis and the process of coding the data was done without trying to fit it into a preexisting coding frame. Thus, we had no pre-identified themes. The analysis was data-driven (Braun & Clarke, 2006). Although extensive notes were taken during the observations, the material analyzed was less rich than in other qualitative methods such as structured interviews. Thus, the elaborate thematic tree was simple, without sub-theme.
- As suggested by the reviewer, to better describe the qualitative study and avoid misunderstanding, the following changes were made:
  - Method section, line 160: “In addition, to complete the evaluation of this early OSCE training program, a qualitative study was conducted via the observation of five of the formative OSCE sessions (the four sessions of one subgroup and one session of another, convenience sampling). Marshall and Rossman defined observation as "the systematic description of events, behaviors, and artifacts in the social setting chosen for study" [13]. It involves active looking, informal interviewing, and writing detailed field notes [14]. Two independent public health researchers trained in qualitative analysis (EL and CE) took extensive notes on students’ behavior and attitudes during observation and did informal interviews with students before and after the sessions. Mixed methods were used in this study for triangulation purposes, which aim to converge results from different methods with the goal of validating interpretations.”
  - Method section, line 191: “Inductive thematic analysis was used for qualitative analysis, we therefore code the data without trying to fit it into a pre-specified coding frame, the analysis was data-driven [15].”
  - Results section, line 253: “The main themes that emerged from the observation analysis were related to stress, the ease and difficulties of the students to act as expected during the exercise, and the reported and observed benefits.”
- Specific answer to the reviewer remarks:

- 1. It is stated on page 12, lines 238-240 that the main themes that emerged from the qualitative analysis were related to....."
Were these the actual main themes? If so this should be made clear. Were there any sub-themes?

- Yes, these were the main themes, without sub-themes, as explained below.

- 2. More detail is needed regarding data collection. How many interviews were conducted with how many students? What questions were asked? In what format were the interview? Where were they conducted? Were they recorded?

- No interview was conducted, as explained above. The qualitative study was conducted via the observation of five of the formative OSCE sessions (method section, line 161).

-3. Likewise how the data analyses was conducted need further explanation. Were the interviews audio recorded and transcribed, for example? If so, by whom? How were the themes decided on?

- No interview was recorded. The observation of the formative OSCE sessions was performed by Drs Enora Le Roux and Clara Eyraud. As frequently used for this qualitative method, the analysis was data-driven, without pre-identified themes (Braun & Clarke, 2006).

-4. It would also be useful for some quotes from the students to be included to illustrate/ support the main themes.

- As no interview was conducted, no quotes were included in the manuscript.
- We hope that the changes made to the manuscript allow a better understanding of the qualitative method used in this study. We are looking forward to a continued discussion for any remaining issues.

References

*Kawulich, Barbara. Participant Observation as a Data Collection Method, Forum: Qualitative Social Research  6 (2), Art. 43 – May 200. DOI:* [*https://doi.org/10.17169/fqs-6.2.466*](https://doi.org/10.17169/fqs-6.2.466)

*Marshall, Catherine & Rossman, Gretchen B. (1989). Designing qualitative research. Newbury Park, CA: Sage. ISBN : 0803931581, 9780803931589*

*Erlandson, David A.; Harris, Edward L.; Skipper, Barbara L. & Allen, Steve D. (1993). Doing naturalistic inquiry: a guide to methods. Newbury Park, CA: Sage. ISBN: 9780803949386*

*DeWalt, Kathleen M. & DeWalt, Billie R. (2002). Participant observation: a guide for fieldworkers. Walnut Creek, CA: AltaMira Press. ISBN : 0759119279*

*Spradley, James P. & McCurdy, David W. (1972). The Cultural Experience. Chicago: Science Research Associates. ISBN : 1577663640*

*Braun V., Clarke V. (2006). Using thematic analysis in psychology.* Qualitative Research in Psychology*, 3, 77–101. doi:10.1191/1478088706qp063oa*
